# Synthesis, Characterization, Electrochemical Properties, and DNA‐Binding Studies of Novel Schiff Base Metal Complexes: Catalysts in Transfer Hydrogenation of D‐Glucose‐Like Aldose Reductase Mimetics

**DOI:** 10.1155/bca/5217456

**Published:** 2026-05-20

**Authors:** Ali Çapan, Abdulkadir Levent, Serhan Uruş, Şerife Yalçın, Mehmet Sönmez

**Affiliations:** ^1^ Vocational School of Higher Education in Nizip, Department of Food Technology, Gaziantep University, Gaziantep, Türkiye, gantep.edu.tr; ^2^ Faculty of Arts and Sciences, Department of Analytical Chemistry, Batman University, Batman, 72100, Türkiye, batman.edu.tr; ^3^ Department of Chemistry, Faculty of Science, Kahramanmaraş Sütçü İmam University, Kahramanmaraş, 46050, Türkiye, ksu.edu.tr; ^4^ Research and Development Centre for University-Industry-Public Relations, Kahramanmaraş Sütçü İmam University, Kahramanmaraş, 46050, Türkiye, ksu.edu.tr; ^5^ Department of Materials Science and Engineering, Graduate School of Natural and Applied Sciences, Kahramanmaraş Sütçü İmam University, Kahramanmaras, 46050, Türkiye, ksu.edu.tr; ^6^ Faculty of Arts and Sciences, Department of Physics, Harran University, Şanlıurfa, 63000, Türkiye, harran.edu.tr; ^7^ Faculty of Arts and Sciences, Department of Chemistry, Gaziantep University, Gaziantep, 27310, Türkiye, gantep.edu.tr

## Abstract

In this study, the Schiff base (E)‐4‐chloro‐2‐(((4‐(phenylamino)phenyl)imino)methyl)phenol (Sadpa), obtained from the condensation reaction of 5‐chloro‐2‐hydroxybenzaldehyde and N^1^‐phenylbenzene‐1,4‐diamine and reported for the first time in the literature, together with its Co(II) and Pd(II) complexes, was synthesized. The structure of the Schiff base was determined by spectroscopic methods such as elemental analysis, FT‐IR, ^1^H/^13^C‐NMR, and XRD, while the structures of the metal complexes were examined in detail using FT‐IR, UV–Vis, MS, thermal, and elemental analysis. Furthermore, the electrochemical behavior of both the Sadpa ligand and the Co(II) and Pd(II) complexes was investigated, as well as their potential interactions with DNA. The double cathodic peak observed in the Sadpa–Co and Sadpa–Pd complexes reveals the coexistence of both ligand and metal‐centered redox processes. It is observed that the Sadpa–Pd(II) complex exhibits more stable and higher affinity binding than the Co(II) complex. This indicates that factors such as coordination geometry and charge density of the metal center play a decisive role in the DNA‐binding behavior. In addition, the catalytic effects of the complexes in the transfer hydrogenation of D‐glucose to D‐sorbitol were investigated. The Sadpa–Pd(II) complex showed the most efficient and selective catalytic activity in the transfer hydrogenation of D‐glucose to D‐sorbitol under micorwave irradiation, with 97.98% selectivity for D‐sorbitol at 96.50% conversion, indicating that it can act as a mimetic of the aldose reductase enzyme. Mannitol was produced as the only by‐product in minor amounts during the catalysis.

## 1. Introduction

Schiff bases, which contain the imine (C=N) group, are the products of condensation reactions between carbonyl compounds and primary amines [1, 2]. These compounds are widely used in coordination chemistry due to their structural diversity, ease of synthesis, and large number of donor atoms [3]. Schiff bases are not only basic organic compounds but also important ligands that can form biologically active complexes through coordination with metal ions [4, 5]. Schiff bases and transition metal complexes are attracting considerable attention due to their versatile coordination chemistry, biological activities [6], and potential as enzyme mimics and DNA targeting agents [7, 8]. Recent studies on the synthesis and structural characterization of these complexes have demonstrated their redox properties, antioxidant [9] and antimicrobial activities [10], and, more importantly, their ability to mimic the activity of aldose reductase [11], an enzyme of critical importance in diabetic complications [12]. Moreover, their interactions with DNA [8], including intercalation and cleavage, have highlighted their potential as promising chemotherapeutic agents [13–15].

Schiff base–metal complexes also exhibit excellent catalytic properties in a variety of reactions, including hydrogenation, hydroxylation, epoxidation, aldol condensation, and oxidation of organic compounds [16]. In addition, imine–metal complexes can be used as catalysts in the ring‐opening polymerization of cyclo‐organic structures [17], the reduction of the carboxyl groups to alcoholic substituents [18], and the alkylation of allylic substrates [19]. Besides, these types of metal complexes, especially cobalt and chromium complexes, exhibit significant effective catalytic activities in the enantioselective catalytic applications [20]. In particular, palladium [21], ruthenium [22, 23], nickel [24, 25], molybdenum, and tungsten complexes display significantly higher catalytic activity in the hydrogenation of unsaturated compounds compared to cobalt and chromium complexes [26–28]. The catalytic hydrogenation of D‐glucose to D‐sorbitol is industrially extremely important due to the million tons of D‐sorbitol used in rising demand for sugar‐free products [29], food additives, pharmaceuticals [14], cosmetics, and raw chemicals in the synthesis of ascorbic acid (C‐vitamin) [16, 28, 30–33].

D‐sorbitol is an important additive widely used in many industrial areas, including pharmaceuticals, cosmetics, food, processed foods, confectionery, toothpaste, and other personal care products, where it functions as a humectant, stabilizer, softener, emulsifier, and bodying agent [34–36]. In addition, D‐sorbitol can be directly or indirectly used in the chemical synthesis of succinic acid, itaconic acid, fumaric acid, levulinic acid, L‐ascorbic acid (vitamin C), isosorbide, lactic acid, ethanol, biohydrocarbons, and several polyols (e.g., glycerol, xylitol) [22, 29, 34, 37]. Conventionally, D‐sorbitol is synthesized on an industrial scale via the catalytic hydrogenation of glucose in batch processes, with an annual production volume of 1.1 million tons using Raney nickel catalysts [38, 39]. Although Raney nickel catalysts provide good catalytic activity, low cost, and excellent sedimentation properties, their tendency to leach and their relatively low selectivity toward D‐sorbitol limit the economic attractiveness of this process [11, 27, 36]. The carbonyl groups of the saccharides can be reduced in the reaction mechanism under hydrogen pressure or transfer of the hydrogen in a primary alcohol with a catalyst containing Ni, Pd, or Pt, Ru‐doped metals, or their cations [40–42]. The use of a hydrogen donor instead of molecular hydrogen has been developed as a greener and more efficient alternative to conventional industrial processes, particularly due to the challenges of hydrogen production and storage safety. In recent years, the synthesis of new catalysts containing different metal complexes (Ni, Pd, Pt, or Ru) has been explored for the production of D‐sorbitol as alternatives to Raney‐type Ni catalysts. Some Ru and Pd complexes have shown higher catalytic activity and sorbitol selectivity than Raney nickel catalysts, with Pd(II) complexes exhibiting the best performance, achieving nearly 98.67% selectivity toward sorbitol [23, 24, 42–44].

Schiff bases derived from 5‐chloro‐2‐hydroxybenzaldehyde, a salicylaldehyde derivative, and N^1^‐phenylbenzene‐1,4‐diamine, an aromatic amine derivative, offer significant potential in liquid crystal, antimicrobial, optical, and biological applications due to their structural and functional properties [45, 46]. 5‐Chloro‐2‐hydroxybenzaldehyde provides a stable and redox‐active coordination environment at metal centers with phenolic O and imine N donors [47, 48], while N^1^‐phenylbenzene‐1,4‐diamine, with its aromatic and conjugated structure, expands the ligand’s *π* system, accelerating electron transfer and enhancing electrocatalytic performance [12, 49]. Metal complexes of these ligands stand out for their strong biological activities compared to the free ligands, particularly their high‐affinity binding to DNA and various binding modes [41, 50, 51].

In this study, a new Schiff base, (E)‐4‐chloro‐2‐(((4‐(phenylamino)phenyl)imino)methyl) phenol (Sadpa) ligand, was synthesized via the condensation of 5‐chloro‐2‐hydroxybenzaldehyde and N^1^‐phenylbenzene‐1,4‐diamine. Its Co(II) and Pd(II) transition metal complexes were prepared using the corresponding acetate salts. The compounds were characterized by elemental analysis, FT‐IR, UV–Vis, ^1^H/^13^C‐NMR, LC‐MS/MS, TGA/DTA, and XRD (for the ligand only). Additionally, their electrochemical behaviors and DNA‐binding affinities were evaluated by voltammetric and spectroscopic methods, while binding energies and target sites were determined by molecular docking studies. Finally, the catalytic effects of the complexes were investigated in the transfer hydrogenation of D‐glucose to D‐sorbitol under microwave irradiation.

## 2. Material and Methods

### 2.1. General

All reagents were of analytical grade and used without further purification. 5‐Chloro‐2‐hydroxybenzaldehyde, N^1^‐phenylbenzene‐1,4‐diamine, Co(CH_3_COO)_2_·4H_2_O, and Pd(CH_3_COO)_2_ were purchased from Sigma‐Aldrich. Elemental analyses were carried out using a Thermo Scientific Flash 2000 instrument. ^1^H NMR and ^13^C NMR spectra were recorded on a Bruker Avance 400 MHz spectrometer. FT‐IR and UV–Vis spectra were obtained using a PerkinElmer Spectrum 100 and PG Instruments T80+, respectively. Crystal data were collected using a Bruker APEX‐II CCD diffractometer. TGA curves were recorded with a STA7300 HITACHI instrument in a nitrogen gas (N_2_) environment. Molar conductivity measurements were performed using an ORION 4 STAR, and magnetic moment measurements were obtained with a Sherwood Scientific instrument. Melting points were determined using a Stuart SMP30 apparatus, and mass spectra were recorded with an LC/MS/MS AB‐Sciex 3200 Q‐Trap. Electrochemical analyses were performed with an Autolab PGSTAT 128N system.

Microwave syntheses were carried out in quartz vessels sealed with Teflon caps using a Milestone StartSynth instrument with temperature control. Catalytic experiments were performed on a Shimadzu HPLC LC‐20AD system equipped with an Inertsil NH_2_ column and RID‐10A detector. Calibration standards of D‐glucose, D‐sorbitol, D‐mannitol, and D‐fructose were prepared in acetonitrile and analyzed by HPLC. External calibration curves were generated using the instrument software. Separations were performed on a Carbosep Coregel Inertsil NH_2_ column (300 × 7.8 mm). All samples and standards were filtered through PVDF 0.45‐μm syringe filters (Teknokroma) prior to injection. Each analysis was completed within 30 min.

### 2.2. Synthesis of Sadpa

**FIGURE 1:**
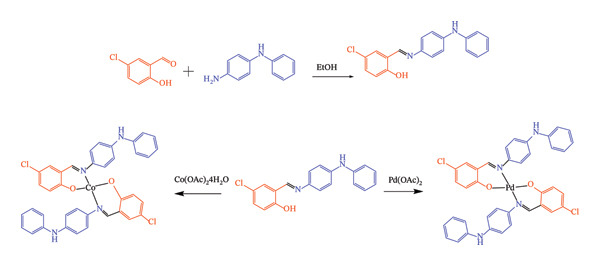
Scheme of formation of Sadpa ligand and metal (II) complexes.

In the synthesis (Figure 1), 10 mmol of 5‐chloro‐2‐hydroxybenzaldehyde was dissolved in 15 mL of ethanol and stirred magnetically. Separately, 10 mmol of N^1^‐phenylbenzene‐1,4‐diamine was dissolved in 15 mL of ethanol and added dropwise to the above solution. The mixture was refluxed with stirring for 3 h, then cooled to room temperature, whereupon yellow crystals formed. The product was isolated by filtration and dried under vacuum. Yield: 80%. M.p. 140°C. Anal. (%) Calc., for C_19_H_15_ClN_2_O (molecular weight: 322.79): C, 70.70; H, 4.68; N, 8.68. Found: C, 69.62; H, 5.104; N, 8.731%. ^1^H NMR (400 MHz, DMSO) *δ* 13.52 (s, 1H, OH), 8.93 (s, 1H, HC=N), and 8.47 (s, 2H, NH), 69–6.89 (ArH). ^13^C NMR (101 MHz, DMSO) *δ* 159.31 (N=CH), 158.36, 144.00, 143.17, 138.48, 132.31, 131.15, 129.71, 123.13, 122.87, 120.91, 118.90, 118.03, 117.10, 116.37. IR, (ATR)v, cm^−1^: *ν* (OH/NH) 3421, *ν* (C‐H)aromatic 3025, *ν* (C=N) 1613, *ν* (C=C) 1558, 1350 (C–O)phenolic *ν*(C‐Cl) 694. UV–Vis (DMF, λmax nm, (Abs.)): 400 (2376), 280 (1555) nm.

### 2.3. Synthesis of the Complexes

The ligand (1 mmol) was dissolved in 10 mL of MeOH. A solution of Co(OAc)_2_4H_2_O or Pd(OAc)_2_ (0.5 mmol) in 10 mL of MeOH was then added dropwise to the reaction mixture, resulting in an immediate color change. After 20 min, precipitation of the metal complex was observed, and the reaction was completed within 2 h. The solid product was collected by filtration, washed with ethanol and ether, and dried (Figure 1).

Co(Sadpa)_2_. Yield: 53%. M.P. 255°C–260°C. Anal. (%) Calc., for C_38_H_28_Cl_2_CoN_4_O_2_ (702.49): C, 64.97; H, 4.02; N, 7.98. Found: C, 64.22; H, 3.98; N 7.84%. IR, (ATR)v, cm^−1^: 3306 (N‐H), 3028 (C‐H)aromatic, 1616 (C=N)azomethine, 1377 (C–O)phenolic, 690 (C–Cl), 524 (M‐N), 470 (M‐O). μeff: 4.26 BM. UV–Vis (DMF, λmax nm, (Abs.)): 400 (0.852), 280 (0.929) nm. ΛM (DMF, 10^−3^ M): 14.75 μS cm^−1^. ESI‐MS (m/z+): 701.40 [M‐H]^+^, 700.40 [M‐2H]^+^.

Pd(Sadpa)_2_. Yield: 50%. M.p. 280°C–290°C. Anal. (%) Calc., for C_38_H_32_Cl_2_N_4_O_4_Pd (749.11): C, 60.86; H, 3.76; N, 7.47. Found: C, 60.51; H, 3.66; N, 7.46%. IR, (ATR)v, cm^−1^: 3310 (N‐H), 3028 (C‐H)aromatic, 1608 (C=N)azomethine, 1377 (C–O), 698 (C–Cl), 534 (M‐N); 464 (M‐O). μeff: 0.08 BM. UV–Vis (DMF, λmax nm, (Abs.)): 400 (0.191), 280 (2339) nm. ΛM (DMF, 10^−3^ M): 13.43 μS cm^−1^. ESI‐MS (m/z+): 747.00 [M‐2H]^+^.

### 2.4. Crystal Study

Single‐crystal X‐ray diffraction data for the compound C_19_H_15_ClN_2_O were collected on a Bruker APEX‐II CCD diffractometer using graphite‐monochromated MoKα radiation at 293 K. The structure was solved by direct methods using SHELXS [52], as implemented in the WINGX program suite. Molecular graphics were prepared with ORTEP‐3 for Windows [53]. The refinement was carried out by the direct methods using SHELXL [54].

### 2.5. Voltammetric Method and DNA‐Interaction Studies

Electrochemical behavior and DNA‐binding properties of the selected compounds were investigated using an Autolab PGSTAT 128N potentiostat/galvanostat system. Measurements were carried out in a conventional three‐electrode cell, consisting of a glassy carbon (GC) working electrode, an Ag/AgCl (3.0 M KCl) reference electrode, and a platinum wire auxiliary electrode. Voltammetric measurements were performed in dimethyl sulfoxide (DMSO) solution containing 0.1 M tetrabutylammonium perchlorate (TBAP). Prior to each measurement, the GC electrode was manually polished with an Al_2_O_3_ suspension, rinsed with distilled water, and dried to ensure reproducible and stable signals. In addition, high‐purity nitrogen gas was bubbled through the solution for approximately 10 min to remove dissolved oxygen, thereby creating an inert atmosphere for the experiments.

### 2.6. DNA Immobilization on the GC Electrode Surface

Double‐stranded DNA (dsDNA) was immobilized onto the GC electrode via electrostatic interaction. For this purpose, a DNA solution (30 mg/L) was prepared in phosphate buffer (PB, pH 7.4) containing 0.02 M NaCl. The electrode was kept in this solution under a constant potential of +0.50 V for 240 s to ensure immobilization of the DNA on the electrode surface. After this process, the electrode was briefly immersed in the supporting electrolyte solution to remove weakly bound molecules on the surface [55, 56].

### 2.7. DNA–Complex Interaction Procedure

DNA‐modified GC electrodes [37, 38] were immersed in PB solution (pH 7.4, 0.02 M NaCl) containing Sadpa and its metal complexes (1 mg/L). The interaction was carried out under open‐circuit conditions (without applying external potential), and binding kinetics were monitored over a contact time of 60 s. This approach was employed to investigate the time‐dependent interaction between DNA and the ligand/complex. Differential pulse voltammetry (DPV) was used to characterize the effect of DNA–complex binding on the electrode surface. Measurements were performed in the potential range of +0.00 to +1.40 V, and the resulting anodic current responses were analyzed to quantitatively evaluate DNA binding. Variations in the voltammetric signals were interpreted as direct electrochemical indicators of DNA–complex interactions.

It has been estimated that this interaction may lead to the formation of complex structures ligand/complex + DNA ↔ ligand/complex–DNA. The binding constant (*K*_*b*_) for these interactions was calculated according to the equation reported in the literature [57]. log(1/[ligand]) = logKb + log*I*_(DNA − ligand)_/[(*I*_DNA_) − (*I*_DNA−ligand_)]. In this equation, *K*_*b*_ represents the binding constant of the complex, IDNA represents the oxidation current of free guanine, and IDNA–ligand represents the guanine current obtained after the interaction of DNA with the ligand.

### 2.8. Investigation of DNA–Complex Interactions With UV‐Visible Spectroscopy

The interactions of the Sadpa ligand and its Co(II) and Pd(II) metal complexes with DNA were investigated in detail using UV‐Visible spectroscopy in PBS buffer (pH 7.04). In these analyses, increasing amounts of DNA (0.5–2.5 mg/L) were incrementally added to a 1 mg/L solution of each compound, and the spectra were recorded. The obtained spectral data were then used to elucidate the binding modes.

### 2.9. Transfer Hydrogenation of D‐Glucose to D‐Sorbitol

The catalytic transfer hydrogenation process was optimized according to our previous studies [11, 27]. Optimum catalyst conditions were established, and a 1:1000 catalyst‐to‐substrate ratio was applied for each reaction. Briefly, 0.01 mmol of the complex was dissolved in 10 mL of isopropyl alcohol (99.4%) in a quartz tube suitable for microwave irradiation. Subsequently, D‐(+)‐glucose monohydrate (10 mmol), dissolved in 15 mL of a solution containing 0.1 mmol of K_2_CO_3_, was added to the reaction vessel. The microwave program in the closed system was set as follows: (i) heating from room temperature to 150°C within 20 min at a maximum microwave power of 600 W, (ii) maintaining the temperature at 150°C for 40 min, and (iii) cooling down to ambient temperature within 15 min. Upon completion of the reaction, the mixture was diluted with an appropriate amount of distilled water, filtered through a 0.45‐μm membrane, and 20 μL of the filtrate was injected into the HPLC system (Table 1). Standard solutions of D‐glucose, D‐mannitol, D‐fructose, and D‐sorbitol were analyzed, and external calibration curves were generated by the software. Conversion and selectivity values in the catalytic activity studies were calculated based on the calibration results for D‐glucose, D‐mannitol, D‐fructose, and D‐sorbitol.

**TABLE 1:** HPLC experimental conditions.

Module	Conditions
Pump	Mobile phase acetonitrile: water (70:30%) Flow rate: 0.5 mL/min
Autosampler	Injection volume: 20 µL
Oven	Temperature: 35°C
Detector	Refractive index (+)
Column	Inertsil NH_2_

## 3. Result and Discussion

### 3.1. Characterization of the Compounds

The Sadpa ligand and its Co(II) and Pd(II) complexes were synthesized for the first time. Analytical and spectroscopic techniques were employed to provide a detailed characterization of the structures of all synthesized compounds. The obtained results were consistent with the proposed configurations of the isolated complexes. The complexes were synthesized by reacting 2 mol of the ligand with 1 mol of the metal ion, resulting in chelation with a 2:1 ligand‐to‐metal stoichiometry, as illustrated in Figure 1. The isolated complexes were insoluble in water and most common organic solvents such as methanol, ethanol, chloroform, dichloromethane, acetone, and acetonitrile, but readily soluble in polar aprotic solvents including DMSO and *N,N*‐dimethylformamide (DMF).

#### 3.1.1. ^1^H NMR and ^13^C NMR Spectra of Sadpa

**FIGURE 2:**
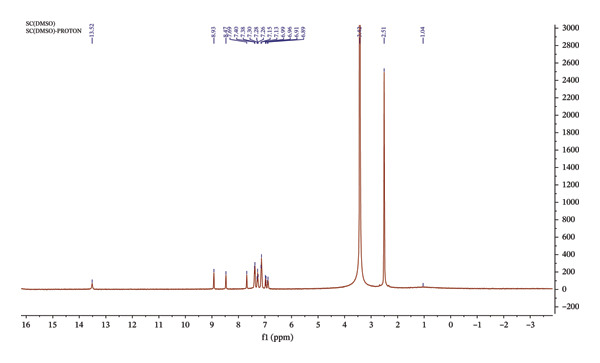
^1^H‐NMR spectrum of Sadpa ligand.

The ^1^H NMR spectrum of Sadpa (Figure 2) exhibits characteristic signals confirming the structural integrity and functional groups of the ligand. The most prominent feature is the broad signal of the phenolic proton, observed around *δ* = 13.52 ppm, attributable to hydrogen‐bonding tendencies of the –OH group [58–60]. The azomethine proton (–CH=N), the key indicator of Schiff base formation, gives a distinct singlet at *δ* = 8.93 ppm. The presence of this signal confirms the successful condensation between the aldehyde and amine moieties, leading to the formation of the imine bond [61, 62]. In the literature, this signal is typically reported in the range of *δ* = 8.5–9.2 ppm for similar compounds [59, 63, 64].

**FIGURE 3:**
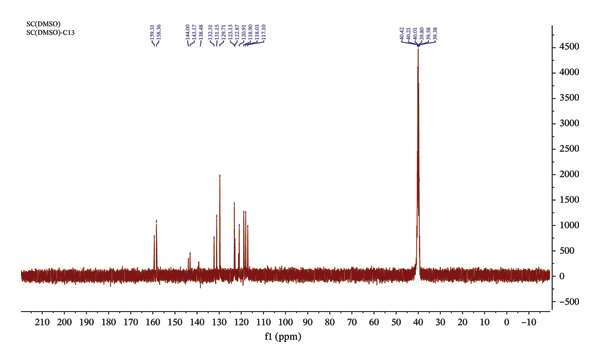
^13^C NMR spectrum of Sadpa ligand.

The ^13^C NMR spectrum of Sadpa (Figure 3) displays characteristic carbon signals that confirm the structural features of the compound and the conjugation between functional groups. Aromatic carbons were generally observed in the *δ* = 116–144 ppm range, reflecting the conjugated nature of the aromatic systems and the influence of different substituents [65, 66]. The carbon directly bonded to the chlorine atom appears at *δ* = 132.31 ppm, indicating an electron‐withdrawing effect of the electronegative chlorine [67, 68]. Carbons adjacent to the NH group exhibit higher chemical shifts (*δ* = 138–144 ppm), attributable to resonance and electron delocalization [59]. Carbon (CH=N) belonging to the azomethine group is located at 158.36 ppm, which showed a high shift value [69, 70]. Additionally, the carbon adjacent to the imine group (C=N–) appears at *δ* = 143.17 ppm, further supporting the presence of the Schiff base moiety [63, 66, 71]. The carbon bonded to the phenolic –OH group resonates at *δ* = 159.31 ppm, consistent with hydrogen‐bonding effects, indicating that the phenolic group is available for coordination. Carbons attached to the aromatic amine group (*δ* = 144.00 and 138.48 ppm) show relatively high chemical shifts due to conjugation effects [58, 61, 67]. Overall, the ^13^C NMR spectrum confirms the structural integrity of the Schiff base, the preservation of the aromatic systems, and the formation of the azomethine group. The observed chemical shifts are consistent with the effects of conjugation, electron density distribution, and substituent influence within the molecule.

#### 3.1.2. XRD Spectral Studies of Sadpa Ligand

**FIGURE 4:**
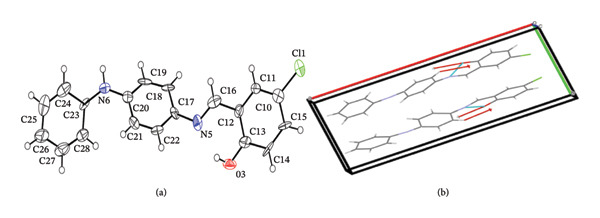
(a) A view of the structure of the Sadpa ligand, showing the atom‐labeling scheme. Displacement ellipsoids are drawn at the 50% probability level in Ortep (b) the crystal packing.

The crystal structure of C_19_H_15_ClN_2_O is depicted in Figure 4(a) (ORTEP representation). The molecule crystallizes in a monoclinic system with space group Pc. The unit cell parameters are determined as *a* = 18.053(8) Å, *b* = 7.159 (3) Å, *c* = 6.115 (3) Å, and *β* = 97.604 (15)°. Detailed crystallographic data and structure refinement information for the compound are summarized in Table 2.

**TABLE 2:** Crystal data and structure refinement of the title compound.

Empirical formula	C_19_H_15_ClN_2_O
Formula weight	322.796
Temperature (K)	273.2
Crystal system	Monoclinic
Space group	Pc
a (Å)	18.053 (8)
b (Å)	7.159 (3)
c (Å)	6.115 (3)
*α* (°)	90
*β* (°)	97.604 (15)
*γ* (°)	90
Volume (Å^3^)	783.3 (6)
Z	2
ρ_calc_g (cm^3^)	1.369
*μ* (mm^−1^)	0.249
F (000)	336.5
Radiation	Mo Kα (*λ* = 0.71073)
2Θ range for data collection (°)	5.7 to 50.98
Index ranges	−22 ≤ *h* ≤ 22, −8 ≤ *k* ≤ 8, −7 ≤ *l* ≤ 7
Reflections collected	13,865
Independent reflections	2880 [Rint = 0.0950, Rsigma = 0.1385]
Data/restraints/parameters	2880/2/2
Goodness‐of‐fit on *F* ^2^	1.032
Final *R* indexes [*I* ≥ 2*σ* (*I*)]	*R*_1_ = 0.1045, wR_2_ = 0.1802
Final *R* indexes [all data]	*R*_1_ = 0.1647, wR_2_ = 0.2037
Largest diff. peak/hole (*e* Å^−3^)	0.74/−0.65

The bond lengths, bond angles, and torsion angles of Sadpa are summarized in Table 3. Examination of the bond lengths shows that the Cl1–C10 bond measures 1.74 Å. The C12–C16, C17–C18, and C17–C22 bond lengths, corresponding to the carbon atoms attached to the azomethine group, are 1.48, 1.38, and 1.39 Å, respectively, consistent with literature reports regarding the influence of the nitrogen atom [72]. The C16=N5 bond length is 1.34 Å, slightly longer than the typical C=N double bond length of 1.28 Å [73]. The N5–C17 bond length is 1.39 Å, which is shorter than the standard C–N single bond length of 1.48 Å. For the secondary amine group, the C19–C20, C20–C21, C23–C24, and C23–C28 bond lengths are 1.32, 1.43, 1.40, and 1.42 Å, respectively, while the C20–N6 and N6–C23 bond lengths are 1.38 and 1.39 Å, respectively. The C13–O3 bond length is observed as 1.32 Å.

**TABLE 3:** Bond lengths, bond angle, and torsion angle of the title compound.

Bond length	(Å)	Bond length	(Å)
Cl1—C10	1.7403	C23—C24	1.4041
O3—C13	1.3244	C12—C16	1.4800
N6—C23	1.3652	C13—C12	1.4350
N6—C20	1.3793	C13—C14	1.3855
N5—C17	1.3854	C22—C21	1.3663
N5—C16	1.3381	C28—C27	1.3338

**Bond angle**	**(°)**	**Bond angle**	**(°)**

C20—N6—C23	132.1	C17—C18—C19	118.3
C16—N5—C17	119.5	C21—C22—C17	123.2
C12—C13—O3	122.6	C19—C20—N6	124.2
C14—C13—O3	118.9	C21—C20—N6	119.5
C14—C13—C12	118.4	C20—C21—C22	118.8
C12—C11—C10	119.4	C22—C17—C18	117.0
C11—C10—Cl1	121.0	C28—C23—N6	122.2
C15—C10—Cl1	117.4	C24—C23—N6	121.9
C18—C17—N5	123.2	C26—C27—C28	124.8
C22—C17—N5	119.8	C12—C16—N5	119.9
C15—C10—C11	121.4	C25—C26—C27	113.4
C20—C19—C18	126.6	C26—C25—C24	124.7

**Torsion angle**	**(°)**	**Torsion angle**	**(°)**

Cl1—C10—C11—C12	177.2	C19—C20—C21—C22	−2.3
Cl1—C10—C15—C14	−179.0	C17—N5—C16—C12	−168.5
O3—C13—C12—C11	178.7	C17—C18—C19—C20	2.6
O3—C13—C12—C16	−2.0	C17—C22—C21—C20	2.7
O3—C13—C14—C15	179.9	C23—N6—C20—C21	−28.6
N6—C23—C28—C27	−174.9	C23—C28—C27—C26	1.8
N6—C23—C24—C25	176.7	C23—C24—C25—C26	−5.5
N6—C20—C19—C18	172.9	C12—C13—C14—C15	2.9
N6—C20—C21—C22	−175.8	C12—C11—C10—C15	2.3
N5—C17—C18—C19	175.4	C28—C23—N6—C20	−29.5
N5—C17—C22—C21	−178.1	C28—C23—C24—C25	3.3
N5—C16—C12—C13	−0.6	C28—C27—C26—C25	−3.3
N5—C16—C12—C11	178.7	C16—N5—C17—C18	39.7
C13—C12—C11—C10	1.9	C16—N5—C17—C22	−142.8
C13—C14—C15—C10	1.1	C16—C12—C13—C14	174.8
C11—C10—C15—C14	−3.9	C18—C19—C20—C21	−0.3
C11—C12—C13—C14	−4.5	C18—C17—C22—C21	−0.4
C10—C11—C12—C16	−177.4	C20—N6—C23—C24	157.6
C19—C18—C17—C22	−2.1	C24—C23—C28—C27	−1.5
C19—C20—N6—C23	158.5	C24—C25—C26—C27	5.2

The carbon atoms exhibit bond angles around 120°, characteristic of sp^2^ hybridization, commonly found in planar regions such as aromatic rings. The C16–N5–C17 bond angle in the azomethine group is approximately 119°, whereas the C20–N6–C23 bond angle in the secondary amine group is around 132°. The molecular planes are slightly twisted and not completely coplanar. The torsion angles C16–N5–C17–N22 and C20–N6–C23–C24 are −142.8° and 157.6°, respectively, and the angle between plane centroids is 167.6°. Detailed information on bond lengths, angles, and torsion angles is provided in Table 3.

The crystal‐packing diagram of the title compound is shown in Figure 4(b). The molecule exhibits intermolecular C–H•••O interactions. A significant intramolecular interaction, N5–H3•••O3, involving the phenolic oxygen atom O3 and nitrogen atom N5, forms a six‐membered S(6) ring, consistent with our previous studies [72]. The CCDC deposition number for this structure is 2422105.

#### 3.1.3. FT‐IR Spectra of the Sadpa Ligand and Its Metal Complexes

In the FT‐IR spectrum of the free ligand (Sadpa), the characteristic *ν* (OH/NH) stretching vibration of the phenolic group is observed at 3421 cm^−1^, while the azomethine *ν* (C=N) stretching vibration appears at 1613 cm^−1^ [58, 61, 74]. Aromatic C=C stretching vibrations appeared at 1558 cm^−1^, consistent with the characteristic aromatic skeletal vibrations typically observed in the 1580–1460 cm^−1^ region. In addition, the phenolic C–O stretching band was detected at 1350 cm^−1^, while the C–Cl stretching vibration appeared around 694 cm^−1^, in good agreement with literature reports for related compounds (Figure S1) [47, 60, 75–78].

The FT‐IR spectra of the Co(II) and Pd(II) complexes exhibit a similar coordination pattern. The secondary amine *ν* (NH) stretching vibration is observed at 3309 cm^−1^ for the Co (Sadpa)_2_ complex, while it appears as a band at 3390 cm^−1^ for the Pd (Sadpa)_2_ complex [67, 74, 76]. The azomethine C=N band changed from 1613 cm^−1^ in the free ligand to 1616 cm^−1^ in Co (Sadpa)_2_ and 1608 cm^−1^ in Pd (Sadpa)_2_, exhibiting a more pronounced peak, indicative of nitrogen atom coordination [77, 79, 80] (Figures S2, S3). Aromatic C=C bands showed slight shifts while maintaining integrity. Although the phenolic *ν* (C–O) band is observed at 1350 cm^−1^ in the free ligand, its shift to the range of 1377 cm^−1^ in the complexes supports the involvement of the phenolate oxygen in metal binding [81–83]. New bands in the 420–560 cm^−1^ region of the isolated metal complexes confirm complex formation, indicating M–N (470–464 cm^−1^) and M–O (524–534 cm^−1^) interactions [77, 79, 80, 84].

#### 3.1.4. UV–Vis Spectra of the Sadpa Ligand and Its Metal Complexes

The UV–Vis spectra of the Sadpa ligand and its Co(II) and Pd(II) complexes (10^−3^ M in DMF) are shown in Figure S4. The free ligand exhibits two intense bands at 280 nm (π ⟶ π^∗^, Abs = 1.555) and 400 nm (*n* ⟶ π^∗^, Abs = 2.376). In the Sadpa–Co complex, these bands appear at 280 nm (0.929) and 400 nm (0.852), and in the Sadpa–Pd complex at 270 nm (2.339) and 400 nm (0.191). These shifts indicate significant changes in transition energies and electron distribution upon complexation, especially in the Pd(II) complex, consistent with similar Schiff base complexes reported in the literature [76, 84, 85].

Additionally, the low‐intensity bands observed in the complexes can be attributed to metal‐centered d–d transitions and/or ligand‐to‐metal charge transfer (LMCT) transitions. Weak d–d bands are masked, especially by ligand‐centered load transfer transition and *n* ⟶ π^∗^ transitions, which are dominant in the UV region. This situation can be explained by the fact that d–d transitions are not completely absent, but rather spectroscopically unresolved due to low molar absorption coefficients and band overlap [74, 76].

#### 3.1.5. Mass Spectroscopy of Co(II) and Pd(II) Complexes of the Sadpa Ligand

ESI–MS analysis confirmed the molecular masses and structural integrity of the complexes [86–88]. The mass spectrum of the cobalt(II) complex exhibits a molecular ion peak at m/z = 701.40 [M‐H]^+^, 700.40 [M‐2H]^+^, which shows excellent agreement with the calculated molecular weight of 702.49 g/mol for the proposed formula C_38_H_28_Cl_2_CoN_4_O_2_ (Figure S5). The fragment at m/z 560.35 is attributed to the partial dissociation of chloride ions and ligand units. Further degradation of the metal–ligand framework gives rise to fragments at m/z 450.25, while the persistent ion observed at m/z 321.30 is assigned to a stable aromatic Schiff base fragment. The fragment at m/z 175.75 is attributed to a smaller organic part.

The mass spectrum of the palladium(II) complex shows a molecular ion peak at m/z = 747.50 [M‐2H]^+^, which corresponds well to the calculated molecular weight of 749.11 g/mol for the proposed formula C_38_H_28_Cl_2_PdN_4_O_2_ (Figure S6). The complex undergoes successive fragmentation initiated by chloride‐ and ligand‐centered dissociation (m/z 609.30), ultimately yielding a preserved fragment at m/z 321.30 associated with the Schiff base framework. In addition, the ions at m/z 249.15 and 166.35 are attributed to aromatic amine fragments.

The isotopic distribution patterns around the molecular ion are characteristic of cobalt and palladium‐containing compounds. The presence of molecular ion peaks in both complexes confirms the proposed 1:2 metal‐to‐ligand stoichiometry and the anhydrous nature of the complexes. The mass spectrometry data, in conjunction with elemental analysis, TGA/DTA, and FT‐IR spectroscopy, unequivocally confirm the successful synthesis of both Co (Sadpa)_2_ and Pd (Sadpa)_2_ complexes.

#### 3.1.6. Magnetic Moment and Conductivity Values of Co(II) and Pd(II) Complexes of Sadpa Ligand

Magnetic susceptibility measurements at 298 ^0^K revealed a *μ*_eff_ of 4.26 B.M. for Co (Sadpa)_2_, suggesting a tetrahedral geometry, consistent with reported tetrahedral Co(II) complexes (4.15–5.1 B.M.). The Pd (Sadpa)_2_ complex exhibited a *μ*_eff_ of 0.06 B.M., in agreement with a square‐planar geometry and paired electrons [13, 63, 84]. Both complexes are nonelectrolytes in DMF, with molar conductivities < 20 μS cm^2^·mol^−1^ [77, 81, 84].

#### 3.1.7. TG Analysis of the Sadpa–Co(II) and Sadpa–Pd(II) Complexes

The thermal stability of the complexes was investigated using TGA. The TGA curves were obtained at a heating rate of 10°C/min in N_2_ atmosphere over the temperature range of 20–850°C (Figure S7, S8). The cobalt(II) complex Co (Sadpa)_2_ remains thermally stable up to approximately 260°C without mass loss, confirming the anhydrous nature of the complex. The main decomposition stage occurs between 260°C and 460°C with rapid mass loss, corresponding to the degradation of the organic part. The mass decreases to 72% at 450°C and continues to decrease gradually to 55% at 730°C. The final residue at 850°C was found to be approximately 49%.

The palladium(II) complex Pd (Sadpa)_2_ exhibits excellent thermal stability up to approximately 280°C, confirming the absence of lattice water or solvent molecules. The main decomposition occurs between 280°C and 400°C, with the mass decreasing to approximately 75% at 400°C. Further gradual decomposition continues, reaching 66% at 600°C and 57% at 850°C.

Both complexes exhibit good thermal stability up to approximately 260°C–280°C, which is advantageous for potential catalytic and biological applications. The complexes are still observed to be undergoing thermal decomposition and mass loss at 850°C. The palladium complex displays slightly higher thermal stability in the initial decomposition region, which can be attributed to stronger Pd–ligand bonds.

### 3.2. Investigation of Electrochemical Behavior of Compounds by Cyclic Voltammetry

#### 3.2.1. Electrochemical Behavior by Cyclic Voltammetry

The electrochemical behavior of Sadpa and its Co(II) and Pd(II) complexes was investigated using CV in DMSO containing 0.1 M TEABF on a GC electrode (Section 2.4). At a scan rate of 100 mV/s (Figure 5(a)), the Sadpa ligand displayed two anodic peaks at +0.191 V (Ia) and +1.131 V (IIa) and a cathodic peak at +0.135 V (IIc), indicating semireversible redox behavior, consistent with literature reports [8, 89, 90]. The Sadpa–Co complex showed anodic peaks at +0.457 V (Ia) and +1.08 V (IIa), and cathodic peaks at +0.2 V (Ic) and −0.308 V (IIc) (Figures 5(a) and 5(c)). The reduction at −0.308 V corresponds to Co(II)/Co(I) transformation, suggesting metal‐centered redox activity. Coordination of Co(II) shifts ligand redox potentials positively, consistent with previous studies [21, 89].

FIGURE 5(a) 100 mV/s voltage scan of Sadpa, Sadpa–Co, and Sadpa–Pd compounds, (b) Sadpa, (c) Sadpa–Co, (d) cyclic voltammetry curves of Sadpa–Pd complex compounds at different voltage scan rates.

**Figure:**
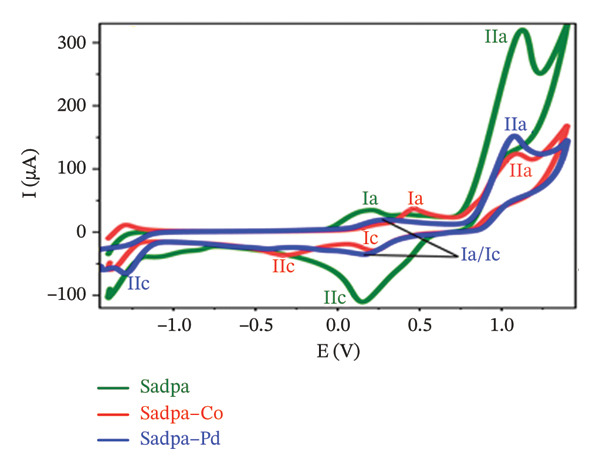
(a)

**Figure:**
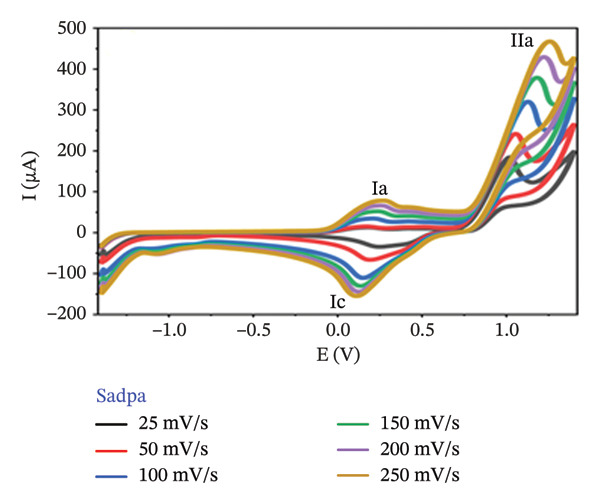
(b)

**Figure:**
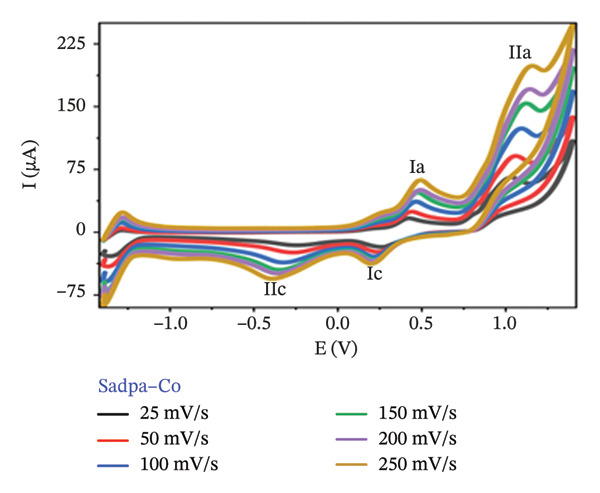
(c)

**Figure:**
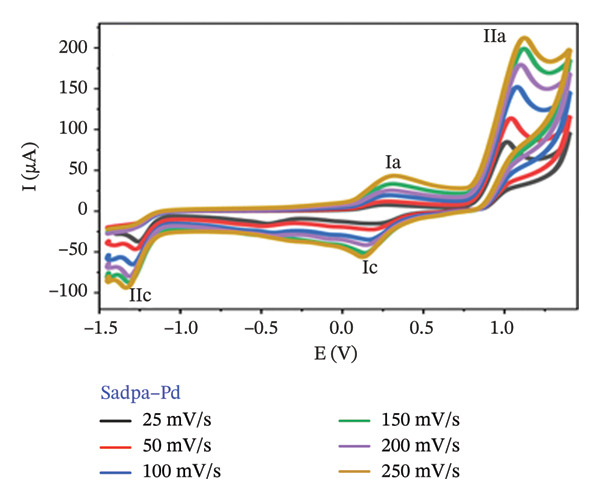
(d)

The Sadpa–Pd complex exhibited anodic peaks at +0.278 V (Ia) and +1.09 V (IIa) and cathodic peaks at +0.154 V (Ic) and −1.284 V (IIc) (Figures 5(a) and 5(d)). The prominent reduction at −1.284 V corresponds to Pd(III)/Pd(II), indicating Pd‐centered redox processes [91, 92].

Figures 5(b), 5(c), and 5(d) presents the CV curves of Sadpa and its complexes obtained at scan rates ranging from 25 to 250 mV/s. The observed increase in current intensity with increasing scan rates in all systems indicates that redox events occur in a diffusion‐controlled manner at the electrode surface. A linear relationship was observed between peak current and scan rate, which is consistent with the classical Randles–Ševčik equation [56, 93]. The double cathodic peak observed in the Sadpa–Co and Sadpa–Pd complexes suggests the coexistence of both ligand‐ and metal‐centered redox processes. This may be related to electronic restructurings induced by metal ions around the ligand. Furthermore, the small shifts observed in the peak potentials with increasing scan rates confirm the semireversible nature of the electrochemical system.

CV curves at scan rates from 25 to 250 mV/s (Figures 5(b), 5(c), 5(d)) show current increases with scan rate, indicating diffusion‐controlled redox events. The linear relationship between peak current and scan rate follows the Randles–Ševčik equation [55, 93, 94]. The double cathodic peaks in Sadpa–Co and Sadpa–Pd complexes reflect coexistence of ligand‐ and metal‐centered redox processes, influenced by electronic restructuring around the metal ions. Small peak potential shifts with increasing scan rate confirm semireversible electrochemical behavior.

#### 3.2.2. Interaction of Sadpa Ligand and Metal Complexes With DNA

The interactions of the Sadpa ligand and its Co(II) and Pd(II) complexes with DNA were systematically investigated using DPV. Experiments were performed on a GC electrode in phosphate buffer (PB, pH 7.4, 0.01 M NaCl), following the procedure described in Section 2.7. The compounds were introduced at different concentrations onto the electrode surface containing immobilized DNA under a constant deposition potential of 0.4 V for 240 s [55, 56]. DPV measurements were recorded after a 60‐s incubation period with the GC/DNA sensor.

Under the applied conditions, neither the Sadpa ligand nor its metal complexes exhibited inherent electroactivity on the bare GC electrode (Figures 6, 7, and 8). Therefore, the binding behavior was evaluated based on changes in the anodic peak current of guanine, which provides a direct measure of DNA interaction. As shown in Figure 6, the DNA‐modified electrode displayed a characteristic oxidation peak for the guanine base at approximately +0.687 V (black curve). Upon addition of Sadpa, the peak shifted to +0.663 V, and the peak current decreased from 0.554 to 0.307 μA, corresponding to a reduction of ∼44.6% (3.22%). This significant decrease suggests that Sadpa interacts with the DNA double helix, thereby restricting guanine access to the electrode surface. Such effects are generally attributed to intercalative or groove‐binding modes [55–57].

FIGURE 6Characterization of Sadpa binding to surface‐immobilized DNA on GC electrode by DPV in PB (pH 7.4, 0.02 M NaCl) media: (a) Overlay of DPV curves recorded pre‐ and postinteraction; (b) quantitative bar chart of guanine peak current attenuation; (c) concentration‐dependent modulation of the guanine signal; (d) binding curve fitted to DPV‐derived data. Experimental conditions: DNA accumulation potential: 0.4 V, DNA accumulation time: 180 s. DNA–Sadpa interaction time: 60 s. DPV parameters: step potential = 0.04 V, modulation amplitude = 0.08 V, modulation time = 0.008 s, interval time = 0.1 s.

**Figure:**
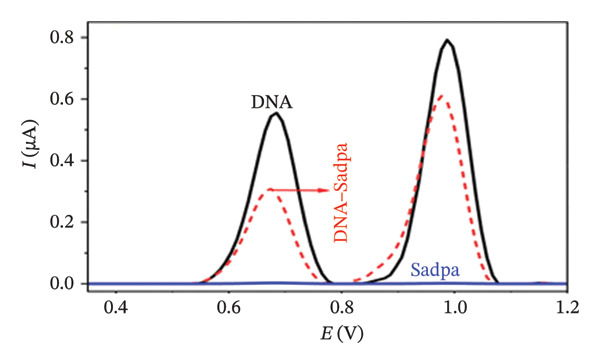
(a)

**Figure:**
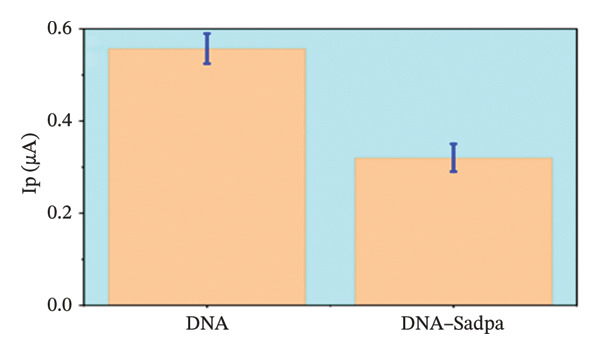
(b)

**Figure:**
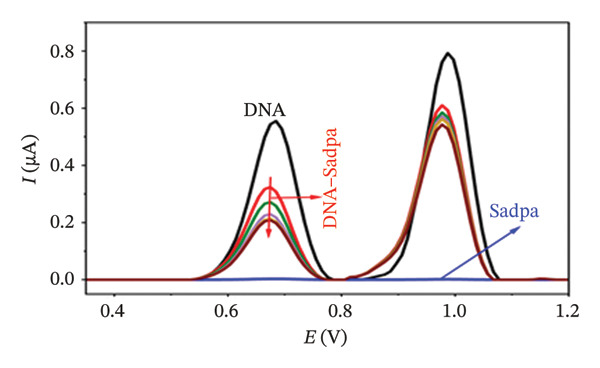
(c)

**Figure:**
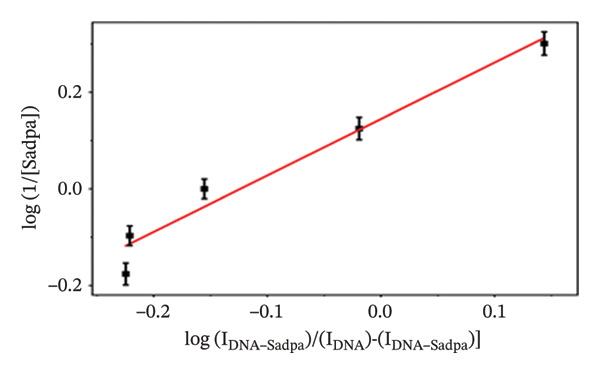
(d)

FIGURE 7Characterization of Sadpa–Co binding to surface‐immobilized DNA on GC electrode by DPV in PB (pH 7.4, 0.02 M NaCl) media: (a) Overlay of DPV curves recorded pre‐ and postinteraction; (b) quantitative bar chart of guanine peak current attenuation; (c) concentration‐dependent modulation of the guanine signal; (d) binding curve fitted to DPV‐derived data. Experimental conditions: DNA accumulation potential: 0.4 V, DNA accumulation time: 180 s. DNA–Sadpa–Co interaction time: 60 s. DPV parameters: step potential = 0.04 V, modulation amplitude = 0.08 V, modulation time = 0.008 s, interval time = 0.1 s.

**Figure:**
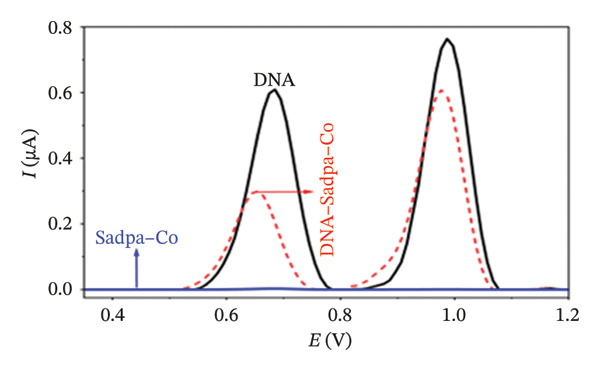
(a)

**Figure:**
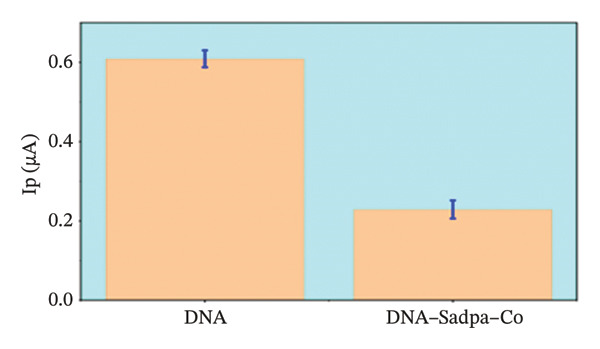
(b)

**Figure:**
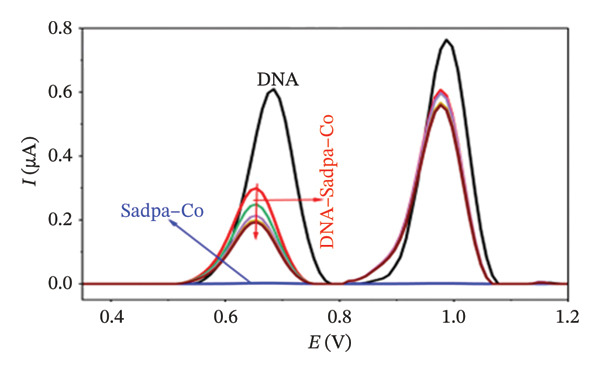
(c)

**Figure:**
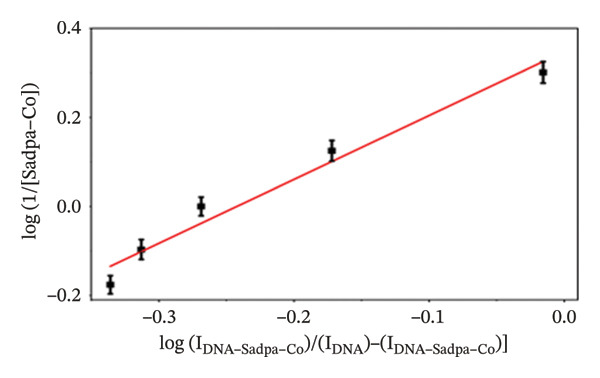
(d)

FIGURE 8Characterization of Sadpa–Pd binding to surface‐immobilized DNA on GC electrode by DPV in PB (pH 7.4, 0.02 M NaCl) media: (a) Overlay of DPV curves recorded pre‐ and postinteraction; (b) quantitative bar chart of guanine peak current attenuation; (c) concentration‐dependent modulation of the guanine signal; (d) binding curve fitted to DPV‐derived data. Experimental conditions: DNA accumulation potential: 0.4 V, DNA accumulation time: 180 s. DNA–Sadpa–Pd interaction time: 60 s. DPV parameters: step potential = 0.04 V, modulation amplitude = 0.08 V, modulation time = 0.008 s, interval time = 0.1 s.

**Figure:**
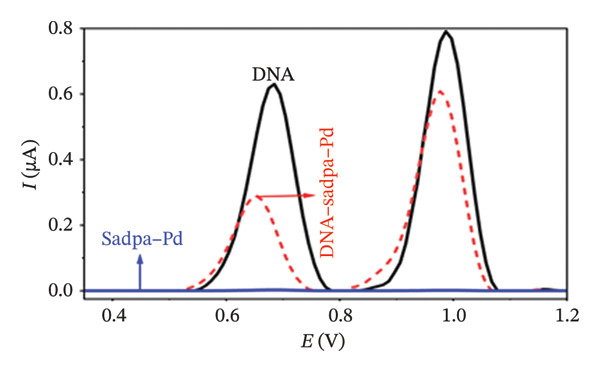
(a)

**Figure:**
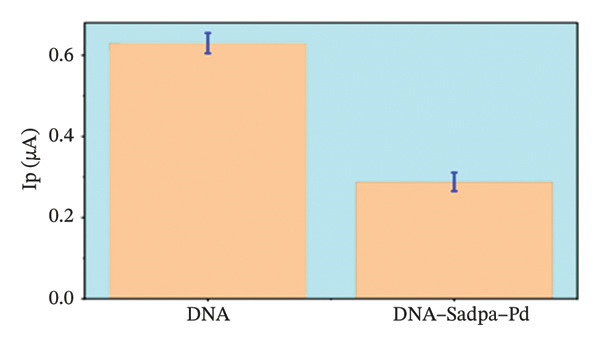
(b)

**Figure:**
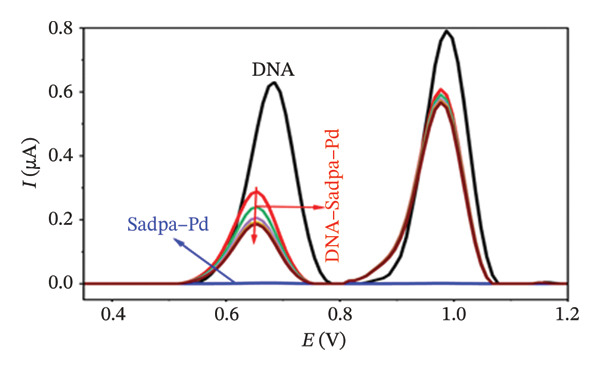
(c)

**Figure:**
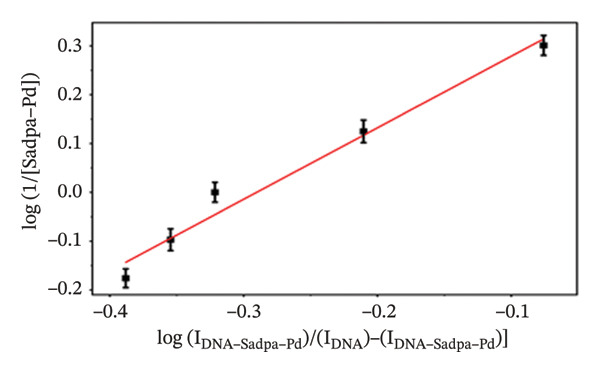
(d)

With increasing concentrations of Sadpa, a significant decrease in the guanine oxidation peak current and a negative shift of approximately 24 mV were observed (Figure 6(c)). This shift suggests that partial conformational changes may occur in the DNA secondary structure due to ligand binding. The binding constant (*K*_*b*_) of Sadpa with DNA was calculated as 1.01 × 10^5^ M^−1^ (Figure 6(d)), indicating a moderate‐to‐high affinity, yet reversible, interaction. This value is consistent with the DNA‐binding capacities reported for aromatic electroactive ligands [55, 95]. These results suggest that Sadpa binds to DNA via intercalative or electrostatic interactions, highlighting its potential for applications in DNA‐targeted biosensors. As illustrated in Figure 8, the GC electrode modified with only DNA displayed a characteristic guanine oxidation peak at +0.684 V. Upon addition of the Sadpa–Co complex, the peak shifted to +0.644 V, and the current decreased from 0.609 to 0.192 μA, corresponding to a reduction of ∼68.5%. These observations indicate that the Sadpa–Co complex strongly binds to DNA and substantially suppresses the electrochemical activity of the nucleobases [96, 97].

As shown in Figure 7(c), increasing concentrations of the Sadpa–Co complex led to a decrease in the guanine oxidation peak current and a negative shift of approximately 40 mV. This behavior suggests an increase in the binding density of the complex to DNA, which may induce conformational changes in the DNA helical structure. The binding constant (*K*_*b*_) of the DNA–Sadpa–Co complex was determined to be 1.42 × 10^5^ M^−1^ (Figure 7(d)), indicating a strong and specific interaction. This high‐affinity binding is relevant for biomolecular recognition, sensor applications, and potential therapeutic use [94, 96, 98].

The DNA–Sadpa–Pd interaction, illustrated in Figure 8(a), shows that the guanine oxidation peak at +0.684 V shifted to +0.640 V upon addition of the Pd complex, while the peak current decreased from 0.630 to 0.185 μA, corresponding to a reduction of approximately 70.6%. These results indicate a strong binding interaction between the Pd complex and DNA [99, 100].

The DPV data for the DNA–Sadpa–Pd system (Figure 8(a)) show that the guanine oxidation peak at +0.684 V shifted to +0.640 V upon addition of the Pd complex, while the peak current decreased from 0.630 to 0.185 μA, corresponding to a reduction of approximately 70.6%. This result indicates a strong interaction between the Pd complex and DNA [99–101].

The interactions of the Sadpa ligand and its Co(II) and Pd(II) complexes with DNA were systematically analyzed using DPV, based on the decrease in guanine oxidation current and shifts in potential. All three compounds exhibited significant binding to DNA. The binding constant of the Sadpa ligand was determined to be moderately high, *K*_*b*_ = 1.01 × 10^5^ M^−1^, whereas the metal complexes exhibited higher binding affinities, with *K*_*b*_ = 1.42 × 10^5^ M^−1^ for Sadpa–Co and *K*_*b*_ = 1.62 × 10^5^ M^−1^ for Sadpa–Pd.

The comparable binding profiles of Sadpa–Co and Sadpa–Pd indicate that the overall DNA affinity is preserved, although the binding mode may vary depending on the metal center. These interactions could involve intercalation, groove binding, or direct metal coordination, consistent with the spectrophotometric data presented in Section 2. These findings suggest that Sadpa derivatives have potential as DNA‐targeted biosensor agents or therapeutic compounds capable of selective and strong DNA interactions.

#### 3.2.3. Investigation of DNA–Complex Interactions by UV‐Visible Spectroscopy

The interactions of Sadpa and its Co(II) and Pd(II) complexes with DNA were further investigated using UV‐Visible spectroscopy in PBS (pH 7.04) according to the protocol described in Section 2.5. Increasing concentrations of DNA (0.5–2.5 mg/L) were added to 1 mg/L solutions of each compound, and the resulting spectra were recorded to evaluate the binding modes.

**FIGURE 9:**
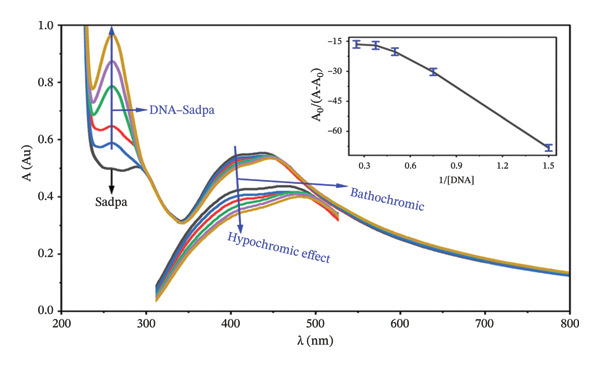
UV–visible spectra of DNA–Sadpa interaction. Inset: binding constant curves of the DNA–Sadpa structure.

The Sadpa ligand exhibited a weak shoulder at 259 nm, a strong peak at 289 nm, and a broad absorbance band at 420 nm (Figure 9). Upon increasing DNA concentrations, the shoulder shifted slightly to 260 nm, accompanied by a pronounced hyperchromic effect. This behavior indicates that the ligand interacts with DNA via external groove binding or electrostatic interactions [55, 56, 102].

Upon interaction with DNA, the 289‐nm peak of Sadpa disappeared completely, and a hypochromic effect was observed in the 420‐nm band, accompanied by a 3‐nm bathochromic shift (420 ⟶ 417 nm). These spectral changes generally indicate an intercalative binding mode. The calculated binding constant, *K*_*b*_ = 1.14 × 10^5^ M^−1^, suggests that Sadpa binds to DNA with moderate affinity, lower than classical intercalators such as ethidium bromide (*K*_*b*_∼10^6^ M^−1^), but still significant [14].

**FIGURE 10:**
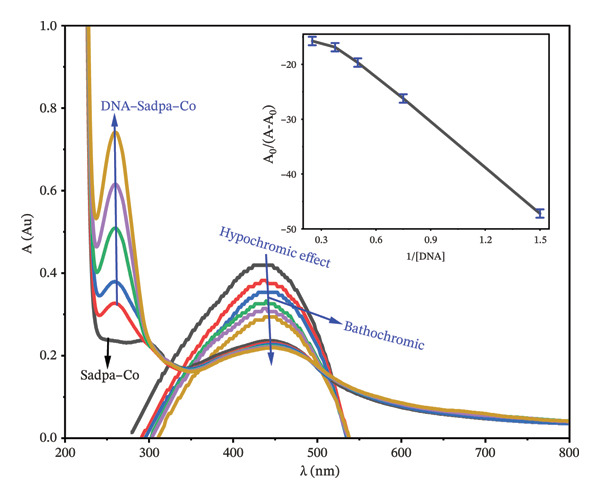
UV‐visible spectra of DNA‐Sadpa‐Co interaction. Inset: binding constant curves of the DNA‐Sadpa‐Co complex structure.

The Sadpa–Co(II) complex exhibited two characteristic absorption bands at 299 nm and 443 nm (Figure 10). Upon addition of DNA, the 299‐nm band disappeared, and a new peak appeared at 260 nm, indicating that the interaction with DNA induces a new electronic environment for the complex. Moreover, the 443‐nm band exhibited a slight bathochromic shift to 440 nm, accompanied by a decrease in intensity, suggesting that the Sadpa–Co(II) complex interacts with DNA via both intercalative and groove‐binding modes.

These observations are in agreement with previously reported DNA‐binding behaviors of Co(II) complexes. For instance, Co(II)–Schiff base complexes have been reported to bind DNA with similar spectral changes and exhibit binding constants around *K*_*b*_∼5.8 × 10^5^ M^−1^ [36, 103]. In the present study, the *K*_*b*_ value of 6.40 × 10^5^ M^−1^ indicates that the Sadpa–Co complex binds strongly to DNA.

**FIGURE 11:**
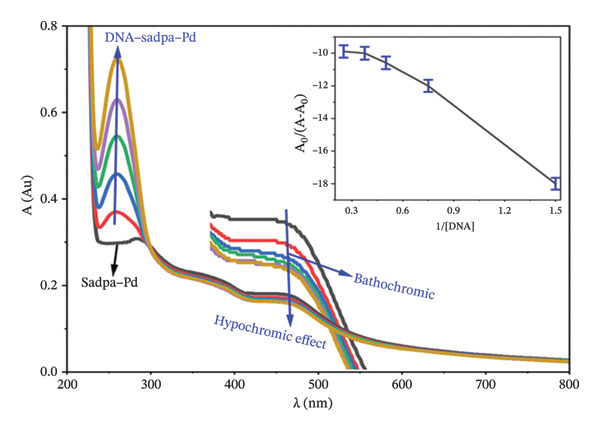
UV–visible spectra of DNA–Sadpa‐Pd interaction. Inset: binding constant curves of the DNA–Sadpa‐Pd complex structure.

The interaction of the Sadpa–Pd(II) complex with DNA produced the most pronounced spectral changes. Initially, three absorption bands were observed at 284, 374, and 459 nm (Figure 11). Upon addition of DNA, the 284‐nm peak disappeared, and a new band appeared at 261 nm. Simultaneously, the 459‐nm band shifted to 461 nm and exhibited a hypochromic effect. These results indicate that the Pd(II) complex interacts strongly with DNA via an intercalative binding mode.

It has been frequently reported in the literature that Pd(II) complexes exhibit high DNA‐binding affinity and potential anticancer activity. For instance, Pd(II) complexes investigated by various researchers bind to DNA via an intercalative mode and show binding constants (*K*_*b*_) in the range of ∼10^5^–10^6^ M^−1^ [104, 105]. Considering the *K*_*b*_ value of 9.89 × 10^5^ M^−1^ obtained in this study, the Sadpa–Pd(II) complex is confirmed as a strong DNA binder. The bathochromic and hypochromic spectral changes observed during DNA–compound interactions indicate that the ligand interacts with the DNA double helix via π–π stacking or intercalation. The DNA‐binding affinity of the Sadpa–Pd(II) complex is comparable to that of classical intercalators reported in the literature [103–105]. Furthermore, the metal center significantly influences DNA interaction, with the Pd(II) complex demonstrating greater stability and higher binding affinity than the Co(II) complex. This suggests that factors such as coordination geometry and metal charge density play a decisive role in DNA‐binding behavior. These findings indicate that the Sadpa–Pd(II) complex could serve as a potential candidate for DNA‐targeted biological applications, particularly in the development of anticancer agents.

### 3.3. Catalytic Activity

Substrate and the hydrogenized sugar alcohols were determined with an optimized method (Figures S7-a–d) as in our previous studies [11, 27], and all the compounds were calibrated in HPLC (Figure S9). If a catalyst would like to be usable and applicable in the catalyst industry, the optimum catalyst:substrate ratio should be 1:1000. Thus, an attempt made to achieve this optimum ratio in order to find the optimum catalyst amount. While decreasing the catalyst below 0.01 mmol such as 0.005 mmol, the conversions and selectivities were calculated below 40%. As reaction time is one of the most important factors in evaluating the efficiency of a catalyst. The optimum reaction time under micorwave power was a total of 1 h, as a very short with efficient conversions and selectivities. When the reaction time was applied shorter than 1 h such as 30 min, conversions and selectivities were calculated around 35%–40%. If longer than 1 h, undesired organic compounds are formed with an increasing conversion to around 90%–95%, whereas decreasing the sorbitol selectivities below 30%. According to the optimization results and our previous studies [11, 27], it was used the optimum catalyst:substrate ratio was 1:1000 in 1 h under microwave power max. 600 W at 150°C.

**FIGURE 12:**
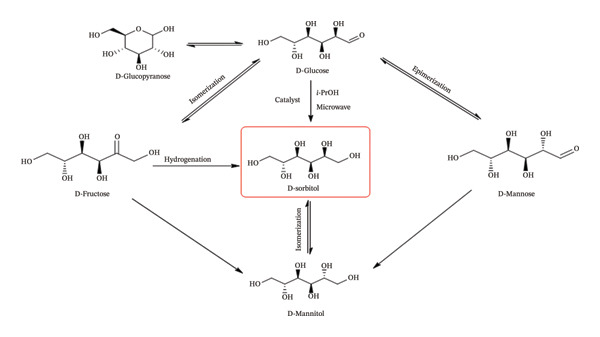
Hydrogenation of D‐glucose [22].

**FIGURE 13:**
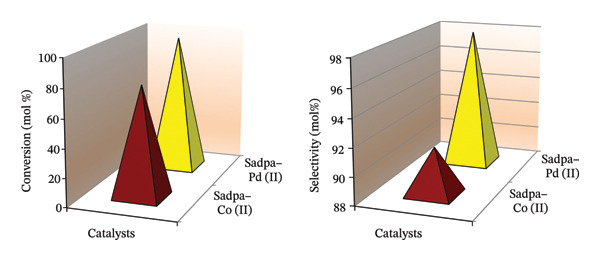
Conversions and selectivities.

Catalysts play a crucial role as “brains” in the selective transfer hydrogenation of D‐glucose to D‐sorbitol (Figure 12). Newly synthesized Pd(II) and Co(II) complexes of the hydroxyphenylimino ligand were employed as catalysts in the microwave‐assisted transfer hydrogenation of D‐glucose using isopropyl alcohol (IPA) as a hydrogen donor. This process conducted in IPA, which acts both as a green, inexpensive, nontoxic, and safe solvent and hydrogen source was completed efficiently within 1 h [11, 27]. Among the catalysts tested, the Pd(II)–Sadpa complex exhibited one of the highest efficiencies and selectivities in the conversion of D‐glucose to D‐sorbitol, surpassing Ru(II)–hydride/phosphine complexes and other reported systems for the transfer hydrogenation of unsaturated organic substrates [34, 43, 106]. The Pd(II) complex, containing the hydroxyphenylimino ligand, demonstrated high efficiency, selectivity, and flexibility through proton transfer in the catalytic mechanism. As a result, the reaction achieved a D‐sorbitol selectivity of 97.98% with a D‐glucose conversion of 96.50% when Pd(II) complex was used as a catalyst, mimicking the activity of the aldose reductase enzyme (Figure S7-e), whereas the Co(II) complex exhibited a selectivity of 91.35% at a conversion of 77.55% (Figure 13, Table 4, Figure S7-f) [22, 34, 103, 107].

**TABLE 4:** Catalysis results.

Catalyst	Conversion	Sorbitol	Mannitol	Selectivity
Sadpa–Co (II)	77.55	70.84	1.52	91.35
Sadpa–Pd (II)	96.50	94.55	1.28	97.98

The Pd(II)–Sadpa complex, with chloro‐substituted aromatic ligands, exhibits excellent electronic and chemical flexibility in the catalytic mechanism. The delocalized π‐electrons of the aromatic ligands, combined with the square‐planar geometry of the Pd(II) center and the Pd(II)/Pd(III) redox transitions, facilitate efficient electron transfer from i‐PrOH (reducing agent) to the metal center and subsequently to the substrate [108].

**FIGURE 14:**
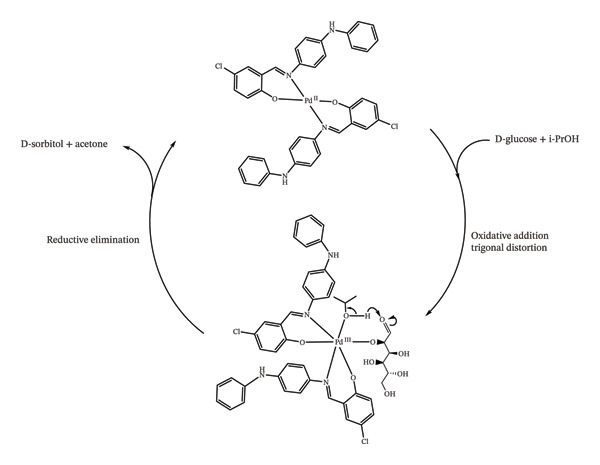
Possible mechanism of transfer hydrogenation of D‐glucose to D‐sorbitol [35, 36].

In the proposed mechanism, the anion in a basic medium coordinates to the metal center via oxidative addition, forming a trigonal distorted Pd(IV) intermediate. This Pd(II)/Pd(III) redox flexibility, observed in cyclic voltammetry (Figure 14), promotes proton transfer from i‐PrOH to the glucose carbonyl group, followed by reductive elimination to regenerate the starting Pd(II) complex [34, 36, 37, 108, 109].

The sterically hindered ligand, coordinated to Pd(II) via strong donor groups such as azomethine and hydroxy, enhances substrate affinity and facilitates proton transfer in the presence of K_2_CO_3_. In addition to geometric and electronic advantages, the π‐back bonding ability of azomethine ligands on chloro‐substituted aromatic rings further enhances catalytic activity [39, 106, 109]. This π‐back bonding, along with steric effects, promotes the formation of a trigonal distorted D‐glucose–Pd(III)–ligand–i‐PrOH precomplex. In this activated intermediate, the sugar anion can efficiently undergo hydrogen transfer from i‐PrOH to its carbonyl group, followed by the release of acetone and D‐sorbitol. Microwave irradiation provides additional excitation, generating positively charged interfaces that facilitate substrate and reducing agent binding (Figure 14) [11, 109]. Moreover, microwave power enhances D‐sorbitol selectivity by minimizing oxidation to gluconolactone and suppressing the isomerization of D‐glucose to D‐mannitol or D‐fructose [27, 104, 110]. Overall, the closed‐system microwave irradiation promotes bond activation via dipole field effects, enabling efficient and homogeneous hydrogen transfer from i‐PrOH to D‐glucose [39, 105, 107, 109].

## 4. Conclusions

In this study, a novel Sadpa was synthesized for the first time via the condensation of 5‐chloro‐2‐hydroxybenzaldehyde with N^1^‐phenylbenzene‐1,4‐diamine. The corresponding Co(II) and Pd(II) complexes of the ligand were successfully prepared using their acetate salts.

The ligand and its Co(II) and Pd(II) complexes were characterized extensively using numerous analytical and spectroscopic methods, including cyclic voltammetric electrochemical studies. All compounds exhibited distinct anodic and cathodic redox signals, and significant shifts in these potentials were observed upon coordination. The dual redox processes observed in the metal complexes indicate the coexistence of both ligand‐ and metal‐centered transformations. Scan rate studies revealed that all systems display diffusion‐controlled and semireversible electrochemical behavior. Notably, the Co(II) complex exhibited a less negative reduction potential, indicating easier reducibility, whereas the Pd(II) complex displayed a more negative reduction potential, attributed to the high‐electron affinity of Pd(II). These results suggest that Sadpa‐based metal complexes have potential applications in electrocatalysis, sensor design, and electrochemical surface modification. Further studies on electrochemical stability, electron transfer kinetics, and biological interactions are warranted.

Spectroscopic studies of DNA interactions revealed that the Sadpa ligand binds to the DNA double helix via π–π stacking or intercalative modes. Furthermore, the metal ion appears to have a significant effect on the interaction with DNA, with the Pd(II) complex exhibiting more stability and higher affinity binding than the Co(II) complex. This suggests that factors such as the coordination geometry and charge density of the metal center play a decisive role in DNA‐binding behavior. These findings suggest that the Sadpa–Pd(II) complex may be a potential candidate for DNA‐targeted biological applications, particularly in the design of anticancer agents.

Finally, the Sadpa–Pd(II) complex, featuring chloro‐substituted hydroxyphenylimino ligands, showed remarkable activity and selectivity in the transfer hydrogenation of D‐glucose to D‐sorbitol. The complex facilitates proton transfer from isopropyl alcohol to the substrate without the need for hydride or phosphine ligands, outperforming many previously reported catalysts. This highlights the Pd(II)–Sadpa complex as an efficient and highly selective catalyst for the conversion of D‐glucose to D‐sorbitol [23, 108].

## Funding

No funding was received for this manuscript.

## Conflicts of Interest

The authors declare no conflicts of interest.

## Supporting Information

Additional supporting information can be found online in the Supporting Information section.

## Supporting information


**Supporting Information** This section provides additional spectral and chromatographic analysis results that support the findings of the study. Figures S1, S2, and S3 present the FT‐IR spectra of the Sadpa ligand and its metal (II) complexes. Figure S4 shows the UV–vis spectra of the ligand and the complexes. Figures S5 and S6 provide the mass spectrometry data of the Co(II) and Pd(II) complexes, respectively. Figures S7 and S8 show the TGA/DTA spectra of the Co(II) and Pd(II) complexes. Figure S9 includes the HPLC chromatograms from the catalytic activity experiments. These chromatograms feature the standards (glucose, fructose, mannitol, and sorbitol), as well as the analyses conducted using the Sadpa–Pd(II) and Sadpa–Co(II) catalysts.

## Data Availability

The authors declare that all data supporting the findings of this study are available within this published article.
