# Supplementary material for: Synthesis, Characterization, Electrochemical Properties, and DNA‐Binding Studies of Novel Schiff Base Metal Complexes: Catalysts in Transfer Hydrogenation of D‐Glucose‐Like Aldose Reductase Mimetics
Source: Bioinorg Chem Appl. 2026 May 20;2026:5217456. doi: 10.1155/bca/5217456 (PMC13189461; doi:10.1155/bca/5217456)
Supplement: Supplementary file 1 — Supporting Information This section provides additional spectral and chromatographic analysis results that support the findings of the study. Figures S1, S2, and S3 present the FT‐IR spectra of the Sadpa ligand and its metal (II) complexes. Figure S4 shows the UV–vis spectra of the ligand and the complexes. Figures S5 and S6 provide the mass spectrometry data of the Co(II) and Pd(II) complexes, respectively. Figures S7 and S8 show the TGA/DTA spectra of the Co(II) and Pd(II) complexes. Figure S9 includes the HPLC chromatograms from the catalytic activity experiments. These chromatograms feature the standards (glucose, fructose, mannitol, and sorbitol), as well as the analyses conducted using the Sadpa–Pd(II) and Sadpa–Co(II) catalysts. [file BCA-2026-5217456-s001.docx]

**Supporting Information**

**Synthesis, Characterization, Electrochemical Properties and DNA Binding Studies of Novel Schiff Base Metal Complexes: Catalysts in Transfer Hydrogenation of D-Glucose Like Aldose Reductase Mimetics**

**Ali Çapan^a*^, Abdulkadir Levent^b^, Serhan Uruş^,c,d,e^, Şerife Yalçın^f^, Mehmet Sönmez^g*^**

^a^Vocational School of Higher Education in Nizip, Department of Food Technology, Gaziantep University, Turkey.

^b^Batman University, Faculty of Arts and Sciences, Department of Analytical Chemistry, 72100, Batman, Turkey

^c^Department of Chemistry, Faculty of Science and Arts, Kahramanmaraş Sütçü İmam University, Kahramanmaraş, 46050, Türkiye

^d^Research and Development Centre for University-Industry-Public Relations, Kahramanmaraş Sütçü İmam University, 46050, Kahramanmaraş, Türkiye

^e^Department of Materials Science and Engineering, Graduate School of Natural and Applied Sciences, Kahramanmaraş Sütçü İmam University, 46050, Kahramanmaras, Türkiye

^f^Harran University, Faculty of Arts and Sciences, Department of Physics, Şanlıurfa, 63000, Türkiye

^g^Gaziantep University, Faculty of Arts and Sciences, Department Chemistry, 27310, Gaziantep, Türkiye

*Corresponding author: Tel: +90 342 3601882; Fax: +90 342 3601032

E-mail adress: [msonmez@gantep.edu.tr](mailto:msonmez@gantep.edu.tr), [alicapan@gantep.edu.tr](mailto:alicapan@gantep.edu.tr)

[Figure S 1. FTIR spectrum of the Sadpa ligand 3](#_Toc219584694)

[Figure S 2. FTIR spectrum of the Sadpa Co(II) metal complex 4](#_Toc219584695)

[Figure S 3. FTIR spectrum of the Sadpa Pd(II) metal complex 5](#_Toc219584696)

[Figure S 4. UV-vis spectra of the Sadpa ligand and metal complexes 6](#_Toc219584697)

[Figure S 5. Mass spectroscopy of the Co(II) metal complex of the Sadpa ligand 7](#_Toc219584698)

[Figure S 6. Mass spectroscopy of the Pd(II) metal complex of the Sadpa ligand 8](#_Toc219584699)

[Figure S 7. Thermogravimetric (TG) analysis of the Co(II)–Sadpa complex. 9](#_Toc219584700)

[Figure S 8. Thermogravimetric (TG) analysis of the Pd(II)–Sadpa complex 10](#_Toc219584701)

[Figure S 9**.** HPLC chromatograms of the catalytic activity experiments. 16](#_Toc219584702)

**FTIR spectrum**

**Figure S 1.** FTIR spectrum of the Sadpa ligand

**Figure S 2.** FTIR spectrum of the Sadpa Co(II) metal complex

**Figure S 3.** FTIR spectrum of the Sadpa Pd(II) metal complex

**UV-vis spectra**


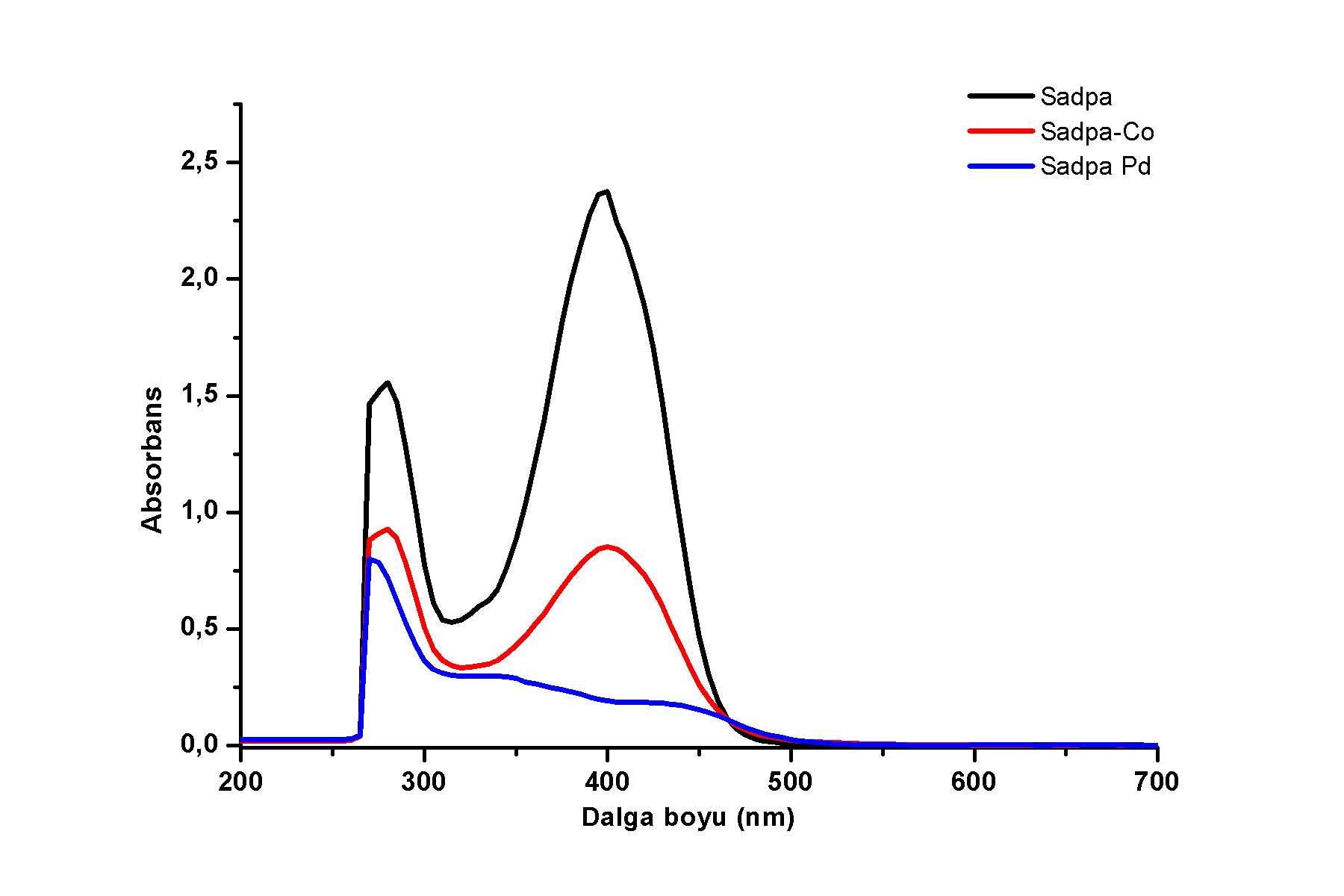


**Figure S 4.** UV-vis spectra of the Sadpa ligand and metal complexes

**Mass spectra**

**Figure S 5.** Mass spectroscopy of the Co(II) metal complex of the Sadpa ligand

**Figure S 6.** Mass spectroscopy of the Pd(II) metal complex of the Sadpa ligand

**Figure S 7.** Thermogravimetric (TG) analysis of the Co(II)–Sadpa complex.

**Figure S 8.** Thermogravimetric (TG) analysis of the Pd(II)–Sadpa complex

**HPLC (High-Performance Liquid Chromatography)**

**(a)**

**(b)**

**(c)**

**(d)**

**(e)**

**(f)**

**Figure S 9.** HPLC chromatograms of the catalytic activity experiments.

1. Chromatogram of glucose standart.
2. Chromatogram of fructose standart.
3. Chromatogram of mannitol standart.
4. Chromatogram of sorbitol standart.
5. Chromatogram of catalysis used Sadpa-Pd(II).
6. Chromatogram of catalysis used Sadpa-Co(II).
